# Patient with Tracheostomy Parasitized in Hospital by Larvae of the Screwworm, *Cochliomyia hominivorax*

**DOI:** 10.1673/031.011.16301

**Published:** 2011-11-30

**Authors:** José A. Batista-da-Silva, Gonzalo E. M. Borja, Margareth M. C. Queiroz

**Affiliations:** ^1^ Universidade Federal Rural do Rio de Janeiro. Rod. BR 465, Km 7, Seropédica (RJ), Brazil; ^2^ Laboratório de Transmissores de Leishmanioses (Setor de Entomologia Médica e Forense) do Instituto Oswaldo Cruz - IOC/FIOCRUZ, RJ, Brazil

**Keywords:** Calliphoridae, ivermectin, myiasis, public health

## Abstract

Myiasis is the infestation of living vertebrates by fly larvae that feed for at least part of their development on the host's dead or living tissues, body substances, or ingested food. The occurrences of traumatic myiasis in humans and animals in urban and rural environments represent serious economic and public health concerns. This study reports a 49-year-old tracheostomized man undergoing chemotherapy treatment who was parasitized in the hospital in São Gonçalo, Rio de Janeiro, Brazil, by larvae of the screwworm, *Cochliomyia hominivorax* (Coquerel) (Diptera: Calliphoridae) in the thoracic cavity.

## Introduction

The Diptera Calliphoridae family is one of the main vectors of myiasis in vertebrates. The larvae of these flies feed on the host's dead or living tissue, body substances, or ingested food during part or all of their immature period (Guimarães and Papavero 1999).

Myiasis is an infestation that affects animals, including humans, causing serious economic and public health problems (Queiroz et al. 2005; Batista-da-Silva et al. 2009). According to Guimarães and Papavero (1999), the type of infestation can be classified by the characteristics of the larva and the damage that it causes: the obligate species parasitize living tissues while the facultative species parasitize necrotic tissues in living individuals. Extensive or chronic wounds or advanced stage wounds with frequent exposure are commonly infested by *Cochliomyia hominivorax* (Coquerel) (Diptera: Calliphoridae). In the literature, myiasis of humans has been associated with low socioeconomic status, alcoholism, mental or neurological diseases, poor personal hygiene, patients with varicose ulcers, diabetes, malnutrition, advanced stages of cancer, pediculosis, immunosuppression, patients with STD, patients with gingivitis, and other lesions in the oral cavity and advanced age (Albernaz 1933; Kaminsky 1993; Verettas et al. 2008). Other factors such as the presence of domestic animals, mendicancy, unhealthy environments, and even debilitated and bedridden patients also contribute to the emergence of new cases (Batista-da-Silva et al. 2009, 2011).

This study presents a case report of a man with tracheostomy undergoing chemotherapy at the National Cancer Institute (INCA-RJ), hospitalized in a public hospital in São Gonçalo, Rio de Janeiro, Brazil, for the treatment of a secondary disease, where he was then parasitized by fly larvae causing obligate myiasis.

## Materials and Methods

The patient was a 49-year-old single man who resided in an urban area of São Gonçalo, Rio de Janeiro, Brazil. He was an ex-alcoholic, bedridden, tracheostomized due to carcinoma of the neck, and was undergoing chemotherapy at the National Cancer Institute-RJ (INCA). During the winter of 2008 (June), he was temporarily hospitalized in a ward of a public hospital in São Gonçalo for the treatment of a secondary disease.

**Figure 1.**
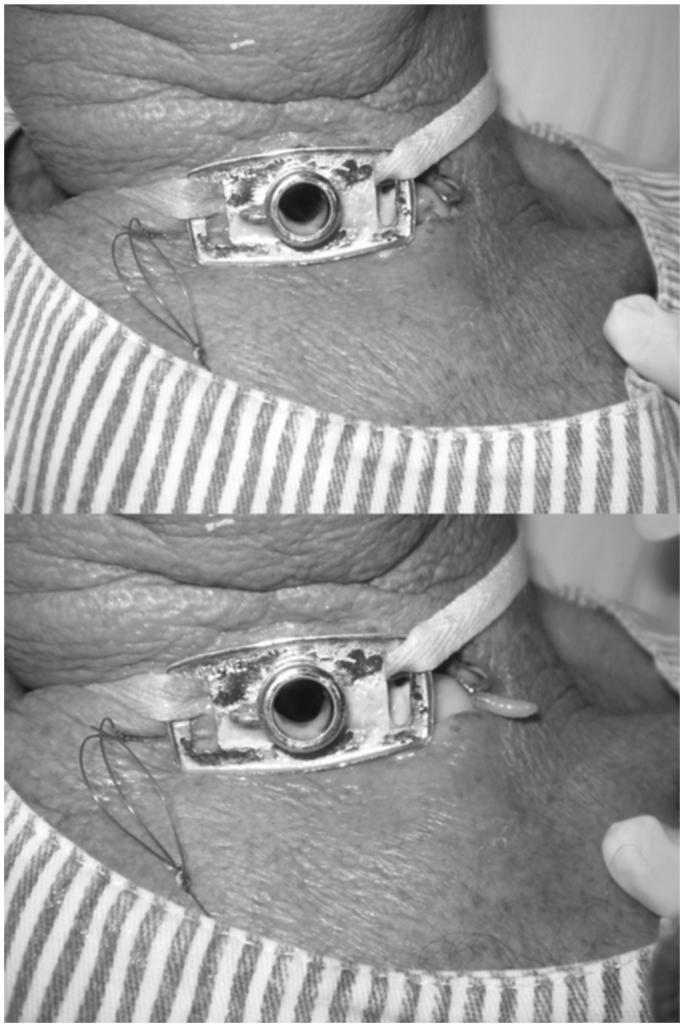
Human myiasis caused by *Cochliomyia hominivorax* in a man with tracheostomy; (A) larva inside the trachea and (B) larva coming out of the trachea. High quality figures are available online.

After eight days of hospitalization, the patient developed an infestation of fly larvae in the thoracic cavity (Figures 1A, 1B). This infestation was the result of oviposition by flies that entered through open unscreened hospital windows.

Medical staff prescribed ivermectin (12 mg/54 kg) in a single oral dose according to description, dosage, and directions to kill the larvae. The patient, who at the time was in a collective ward, was moved to a single room with air-conditioning (21 °C). A total of 32 larvae (third instar) were collected and placed in individual pots and covered with cotton under environmental conditions in a ventilated area to wait for adult emergence. After emergence, adults were sent to the Laboratory of Leishmaniasis Transmitters (Department of Medical and Forensic Entomology) at the Institute Oswaldo Cruz IOC / FIOCRUZ, RJ, Brazil, to be identified in accordance with the key to genera and species (Mello 2003). Temperature and RH (http://satelite.cptec.inpe.br 2008) were collected for twelve days; mean and standard deviations were calculated for the same data during the pupal stage until the emergence of the adults.

## Results

All 32 larvae that were collected from the patient, the bed, and off the floor of the ward were in the third instar, and all had pupated. During this stage, the pupae, under environmental conditions in a ventilated area, were observed daily to see if the variables (temperature and RH) would interfere with the pupal stage or whether there would be any delayed effects caused by the ivermectin on the insects. The total pupation period lasted 12 days for all larvae, with an average temperature of 19.9 — 3.21 °C and 82 — 13.95% RH. All individuals were identified as belonging to species *C. hominivorax*.

The use of ivermectin to kill third instar larvae in the patient did not interfere in its life cycle; however, the lower winter temperatures increased the pupal stage, which normally lasts an average of eight days (Neves et al. 2000). In contrast, the average RH was favorable to the development of larvae and emergence of adult flies.

## Discussion

The patient was undergoing chemotherapy treatment for cancer. Complete laryngectomy (removal of the larynx) results in the loss of physiological voice and definitive tracheostomy (artificial opening in the trachea below the larynx). As preservation of voice quality is a top priority, sometimes radiotherapy is used first, leaving surgery until later if radiotherapy is not sufficient to control the tumor (INCA 2010).

Open wounds and bodily orifices that emit odors of natural secretions are major factors in susceptibility to myiasis (Batista-da-Silva et al. 2011), as they provide a favorable environment for the attraction and oviposition of flies. A bedridden patient is possibly at a higher risk for infestation by fly larvae, a suggestion supported by Smith and Clevenger (1986), who reported that the risk of contracting myiasis increases when the patient is immobile or debilitated. Considering that the patient reported in this study presented an infestation by third instar fly larvae on the eighth day of hospitalization, it is possible that the infestation occurred on the third (72 hours) or fourth day (96 hours) after hospitalization. This provides strong confirmatory support for the results of Hira et al. (2004), where two patients in Kuwait had an infestation more than three days after hospitalization, and according to these authors this case may be considered nosocomial (hospital-acquired) by definition.

Myiases caused by several species of fly larvae and acquired inside the hospital (nosocomial) have been reported in different parts of the world. Smith and Clevenger (1986) reported the occurrence of fly maggots in the nasal cavity of an unconscious 64-year-old man who had been admitted to hospital 18 days earlier in a diabetic hyperosmolar coma. The larvae were identified as *C. macellaria*, an organism commonly associated with myiasis in the United States (Smith and Clevenger 1986). Hira et al. (2004) reported two cases of facultative myiasis in a hospital in Kuwait City; one a case of nasopharyngeal myiasis caused by *Lucilia sericata* and the other a cutaneous myiasis caused by *Megaselia scalaris.* Hardy (1951) also reported larvae of the *M. scalaris* invading a one-year-old surgical site on the chest of a patient in Hawaii.

The use of ivermectin to kill the larvae has been reported by several authors, and depending on its application (usage) it results in a significant reduction of larvae in infested wounds. Ivermectin is a semi-synthetic antibiotic isolated from *Streptomyces avermitilis* (Shinohara et al. 2004). Its use is well-documented in large animals for the control of gastrointestinal and lung parasites, as well as to control infestations of fly larvae. Ivermectin blocks the nerve impulses in the nerve endings by releasing gamma-aminobutyric acid, binding to receptors and causing paralysis and death of larvae (Campbell 1985). Although the literature points out the efficiency of ivermectin in the treatment of myiasis caused by larvae of *C. hominivorax*, this unique case reports an unsuccessful treatment with ivermectin of a cancer patient with a third instar larvae infestation. It is possible that ivermectin might be effective against first and second instars and much less effective against more mature third instar larvae.

According to Graham (1985), early results suggested that ivermectin may be ineffective against *C. hominivorax*, but more recent studies demonstrated that it can produce a significant reduction in the incidence of navel and scrotal myiasis due to *C. hominivorax* (Benitez et al. 1997; Lombardero et al. 1999). There has been an increasing number of publications reporting that a subcutaneous injection of doramectin (200 µg/kg) was up to 100% effective as a *C. hominivorax* prophylactic, preventing infestation of artificial wounds, umbilical or castration wounds of calves, and infestation of post-parturient cows for up to 12–14 days post-treatment (Moya-Borja et al. 1997; Anziani et al. 2000). Caproni et al. (1998) reported a persistent efficacy of doramectin and ivermectin in the prevention of natural *C. hominivorax* infestations in cattle castrated 10 days after treatment. The study was conducted in Brazil to compare the persistent efficacy of a single subcutaneous injection of doramectin and ivermectin at a dose rate of 200 µg/kg in the prevention of myiasis caused by *C. hominivorax* larvae in cattle castrated 10 days after treatment and exposed to natural infection in the field. Doramectin had an efficacy higher than 90% and ivermectina had an efficacy of less than 50% (Caproni et al. 1998). Anziani et al. (2000) showed that doramectin provided a reduction in myiasis of 90.9 and 83.3% at 12 and 15 days after induced infestations of *C. hominivorax* in calves, respectively, compared to the saline control treatment (*p* < 0.01). In contrast, there were no significant differences in the number of calves with myiasis between those treated with the ivermectin formulations or the saline control.

Besides the fact that ivermectin had no effect on third instar larvae observed in this work, another important aspect observed here is the emergence of all adult flies, demonstrating the absence of any delaying effect of ivermectin on the *C. hominivorax* pupae. Only the low winter temperatures may have caused an increase in the pupal stage. The mean temperature was 19.9 — 3.21 °C with 100% emergence in 12 days, which was very similar to data obtained by Elwaer and Elowni (1991), where pupation occurred at a temperature of ∼ 20 °C, and had 94% emergence under controlled conditions.

Since the patient had no contact with alcohol, tobacco, or antibiotics during hospitalization for treatment of a secondary disease, it is possible that treatment with ivermectin is inefficient against third instar larvae of *C. hominivorax*.

The occurrence of a case of myiasis caused by *C. hominivorax* acquired inside the hospital (nosocomial) indicates a degree of neglect of the medical service and ignorance of the medical staff on how to control this parasite in a hospital environment. The primary factor for infestation was from flies that entered through the open, unscreened window. To avoid similar cases in future, it is imperative that windows screens be used in hospital settings.
